# Effects of targeting the transcription factors Ikaros and Aiolos on B cell activation and differentiation in systemic lupus erythematosus

**DOI:** 10.1136/lupus-2020-000445

**Published:** 2021-03-16

**Authors:** Felice Rivellese, Sotiria Manou-Stathopoulou, Daniele Mauro, Katriona Goldmann, Debasish Pyne, Ravindra Rajakariar, Patrick Gordon, Peter Schafer, Michele Bombardieri, Costantino Pitzalis, Myles J Lewis

**Affiliations:** 1 Centre for Experimental Medicine and Rheumatology, William Harvey Research Institute, Barts and The London School of Medicine and Dentistry, Queen Mary University of London, London, UK; 2 Rheumatology Department, Barts Health NHS Trust, London, UK; 3 Renal Department, Barts Health NHS Trust, London, UK; 4 Rheumatology Department, King's College London, London, UK; 5 Translational Medicine Department, Bristol-Myers Squibb Co, Princeton, New Jersey, USA

**Keywords:** lupus erythematosus, systemic, B-lymphocytes, autoimmune diseases

## Abstract

**Objective:**

To evaluate the effects of targeting Ikaros and Aiolos by cereblon modulator iberdomide on the activation and differentiation of B-cells from patients with systemic lupus erythematosus (SLE).

**Methods:**

CD19^+^ B-cells isolated from the peripheral blood of patients with SLE (n=41) were cultured with TLR7 ligand resiquimod ±IFNα together with iberdomide or control from day 0 (n=16). Additionally, in vitro B-cell differentiation was induced by stimulation with IL-2/IL-10/IL-15/CD40L/resiquimod with iberdomide or control, given at day 0 or at day 4. At day 5, immunoglobulins were measured by ELISA and cells analysed by flow cytometry. RNA-Seq was performed on fluorescence-activated cell-sorted CD27^-^IgD^+^ naïve-B-cells and CD20^low^CD27^+^CD38^+^ plasmablasts to investigate the transcriptional consequences of iberdomide.

**Results:**

Iberdomide significantly inhibited the TLR7 and IFNα-mediated production of immunoglobulins from SLE B-cells and the production of antinuclear antibodies as well as significantly reducing the number of CD27^+^CD38^+^ plasmablasts (0.3±0.18, vehicle 1.01±0.56, p=0.011) and CD138^+^ plasma cells (0.12±0.06, vehicle 0.28±0.02, p=0.03). Additionally, treatment with iberdomide from day 0 significantly inhibited the differentiation of SLE B-cells into plasmablasts (6.4±13.5 vs vehicle 34.9±20.1, p=0.013) and antibody production. When given at later stages of differentiation, iberdomide did not affect the numbers of plasmablasts or the production of antibodies; however, it induced a significant modulation of gene expression involving *IKZF1* and *IKZF3* transcriptional programmes in both naïve B-cells and plasmablasts (400 and 461 differentially modulated genes, respectively, false discovery rate<0.05).

**Conclusion:**

These results demonstrate the relevance of Ikaros and Aiolos as therapeutic targets in SLE due to their ability to modulate B cell activation and differentiation downstream of TLR7.

**Key messages**

**What is already known about this subject?**

- The transcription factors Ikaros and Aiolos, which are critical for B cell differentiation, are implicated in systemic lupus erythematosus (SLE) pathogenesis.
- Targeting Ikaros and Aiolos using the cereblon modulator iberdomide has been proposed as a promising therapeutic agent.

**What does this study add?**

- Targeting Ikaros and Aiolos with iberdomide inhibits the TLR7-mediated differentiation of plasmablasts in human SLE.
- Iberdomide inhibits TLR7 and interferon driven autoantibody production by SLE autoreactive B cells.
- RNA-sequencing was used to detect differentially expressed genes in iberdomide-treated differentiated SLE plasmablasts, thus identifying genes which are most directly under the control of Ikaros and Aiolos.

**How might this impact on clinical practice or future developments?**

- This study provides strong evidence that therapeutic targeting of Ikaros and Aiolos can ameliorate key pathogenic processes in human SLE.

## Introduction

The Ikaros family of zinc-fingers (IKZF) proteins are transcription factors critical for the development of key haemopoietic stem cell lineages and their maturation into effector cells.^1^ IKZF1 (Ikaros) is an essential regulator of common lymphoid progenitor (CLP) stem cells. *Ikzf1*-deficient mice lack CLP and fail to generate mature B/T lymphocytes, natural killer and dendritic cells.^2 3^ IKZF1 also acts as a checkpoint at the pro-B cell to pre-B cell transition stage.^4 5^ In contrast, IKZF3 (Aiolos) is necessary for memory B cell and plasma cell formation and *Ikzf3* deficient mice lack both of these mature B cell populations.^6 7^

*IKZF1*, *IKZF3* and more recently *IKZF2* (Helios) have been identified as susceptibility loci in systemic lupus erythematosus (SLE) in large-scale genome-wide associated studies.^8–13^ *IKZF1* polymorphism rs4917014 was identified as a trans-expression quantitative trait locus, driving upregulation of type 1 IFN genes and downregulation of complement genes.^14^ Ikaros has also been shown to influence TLR7 signalling,^15^ representing another link with SLE pathogenesis.^16^

Ikaros and Aiolos have emerged as the therapeutic targets of the immunomodulatory drugs thalidomide and analogues such as lenalidomide, which act as agonists for the ubiquitination E3 ligase complex cereblon,^17^ thus inducing ubiquitination and proteasomal degradation of Ikaros and Aiolos.^18–20^ Thalidomide and its analogues are well established as treatment options for multiple myeloma.^21^ Lenalidomide inhibits plasma cells differentiation from healthy donors in vitro^22^ and has been found to be effective in cases of treatment-refractory cutaneous manifestations of SLE.^23–25^ More recently, a novel cereblon ligand has been developed, iberdomide, which binds with higher affinity than thalidomide and other analogues, resulting in greater Ikaros/Aiolos degradation.^26^

In preliminary studies, iberdomide reduced Ikaros and Aiolos expression in SLE B cells, while inhibiting antibody production and differentiation of B cells into plasmablasts.^27^ Iberdomide has undergone a phase I study, which explored the effects of oral administration in healthy volunteers and confirmed the reduction of Ikaros and Aiolos at protein level in B cells, T cells and monocytes.^28^ Iberdomide also inhibited anti-dsDNA and antiphospholipid antibody production ex vivo from SLE mononuclear cells. Iberdomide reduced absolute B cell counts, augmented IL-2 production from T cells and inhibited IL-1β production in response to proinflammatory stimuli, underpinning its further development for the treatment of SLE, with an ongoing phase II trial (Clinical-Trials.gov Identifier: NCT02185040).

In this study, we set out to investigate the effects of targeting Ikaros and Aiolos with iberdomide on peripheral blood B cells from patients with SLE, specifically on (i) TLR7 and IFN mediated B cell activation and differentiation, (ii) plasmablast/plasma cell differentiation and (iii) transcriptional programmes underlying B cell differentiation.

## Materials and methods

### Patient recruitment

Peripheral blood and clinical demographics were collected from patients with a diagnosis of SLE from Barts Health NHS Trust and Kings College Hospital NHS Trust. All study participants provided written informed consent at time of sample collection and fulfilled the 2012 updated American College of Rheumatology classification criteria ACR criteria for SLE.^29^ Patient characteristics are summarised in table 1. Sample collection from patients and subsequent analysis were approved by UK local ethical committee (REC reference 17/WS/0172). Patients or the public were not involved in the design, conduct, reporting or dissemination plans of the research.

**Table 1:** Summary of patient characteristics (n=41)

Age, years mean (SD), range	47 (13)	21–72
Gender (female)	81%	
ANA+	86%	
dsDNA+	62%	
APS+	19%	
Sm+	14%	
Skin involvement*	60%	
Arthritis	60%	
Serositis	15%	
Renal disease	10%	
Leucopaenia	25%	
Thrombocytopaenia	5%	
Hydroxychloroquine	86%	
Steroids	37.5%	

*Including malar rash, photosensitivity and discoid lupus.

### Blood processing and B cell isolation

Peripheral blood mononuclear cells were isolated from peripheral blood using gradient separation with Lymphoprep (Alere), with RBC lysis via incubation in ammonium chloride (RBC-lysis buffer, Stemcell) for 5 min on ice. CD19^+^ cells were isolated via negative magnetic selection with EasySep Human B Cell Isolation Kit (Stemcell). Purity (>95%) of isolated B cells was verified by flow cytometry. For selected experiments, 1–2×10^5^ B cells at baseline were stored in RNAprotect Cell Reagent (QIAGEN) and frozen at −80°C for subsequent RNA extraction.

### B cell culture and TLR7/IFN stimulation

Human B cells freshly isolated from patients with SLE were resuspended at concentration 0.5×10^6^ cells/mL in Iscove’s modified Dulbecco’s medium (IMDM) with 10% fetal calf serum (FCS), 1% antibiotic-antimycotic (Gibco) and 3 µM synthetic TLR7 agonist Resiquimod (R848, Invivogen) with/without 1000 IU IFNα (Enzo), together with iberdomide 1, 10 or 100 nM or vehicle control. At 5 days, cells were harvested for fluorescence-activated cell sorting (FACS) analysis, and supernatants stored for ELISA.

### B cell culture and plasmablast differentiation

Human B cells freshly isolated from SLE patients were resuspended and cultured using a modified plasmablast differentiation protocol^30^ with HA-sCD40L (R&D) at 50 ng/mL cross-linked with 1 µg/mL anti-HA IgG (R&D), IL-2 at 20 U/mL, IL-10 at 50 ng/mL (Peprotech), IL-15 at 10 ng/mL (Peprotech) and TLR7 ligand R848 at 3 µM (Invivogen). Iberdomide (10 nM) or control vehicle was added at two timepoints, at either day 0 or 18 hours prior to harvest on day 4. After 5 days, cells were harvested for FACS, and supernatants stored for ELISA.

### Flow cytometry and fluorescence activated cell sorting

Cultured B cells were characterised by flow cytometry on BD LSR Fortessa II with Live/Dead staining with Zombie NIR Dye (Biolegend), Fc blocking using Human Truestain FcX (Biolegend) and incubation with antibodies listed in online supplemental table 1). Samples undergoing plasmablast differentiation were cell sorted on BD FACSAria II into IgD^+^CD27^-^ naïve B cells and CD20^low^CD27^+^CD38^+^ plasmablasts. Immediately after sorting, cells were resuspended in RNAprotect Cell Reagent (QIAGEN) and frozen at −80°C for RNA extraction. Data were analysed using Flowjo software V10.

10.1136/lupus-2020-000445.supp1 Supplementary data

### ANA and immunoglobulin measurement

To measure antinuclear antibodies (ANA), 30 µL of neat supernatant was added to Hep-2 precoated microscope slides (BioDiagnostics). After washing in phosphate-buffered saline (PBS), bound immunoglobulins were detected with AF488 antihuman-IgG antibody (Invitrogen). Slides were read on an Olympus BX61 microscope interface with CellSense software. IgG and IgM were measured using total IgG and IgM ELISA Kits (Bethyl).

### RNA sequencing

RNA was extracted using RNeasy Micro Kit (Qiagen) and concentration quantified on NanoDrop 2000c (ThermoFisher). RNA sequencing libraries were prepared using the NEBNext Ultra RNA Library Prep Kit for Illumina, validated on Agilent TapeStation and quantified using Qubit 2.0 Fluorometer (Invitrogen) and qPCR (KAPA). Libraries were sequenced on Illumina HiSeq 4000, using 2×150 bp paired end configuration, 50 million reads/sample.

### RNA-seq data processing and analysis

Paired-end RNA-seq samples of 150 base pairs were trimmed to remove the Illumina adaptors using bbduk from BBMap package V.37.93. Transcripts were then quantified using Salmon version 0.13.1 with an index generated from Gencode release 29 transcriptome. Tximport version 1.13.10 was used to aggregate transcript level expression data to genes, and differential gene expression analysis was performed using DESeq2 V.1.25.9. P values were false discovery rate adjusted using Benjamini-Hochberg method. Genes regulated by Ikaros and Aiolos were identified via the Harmonizome website^31^ from ENCODE,^32^ CHEA Transcription Factor Targets,^33^ the Pathway Commons Protein-Protein Interactions (http://www.pathwaycommons.org/) and published literature^5 14 34^ (online supplemental table 2).

10.1136/lupus-2020-000445.supp2 Supplementary data

## Results

### Iberdomide inhibits TLR7 and IFNα-induced activation of SLE B cells and abrogates autoantibody production

**Figure 1:**
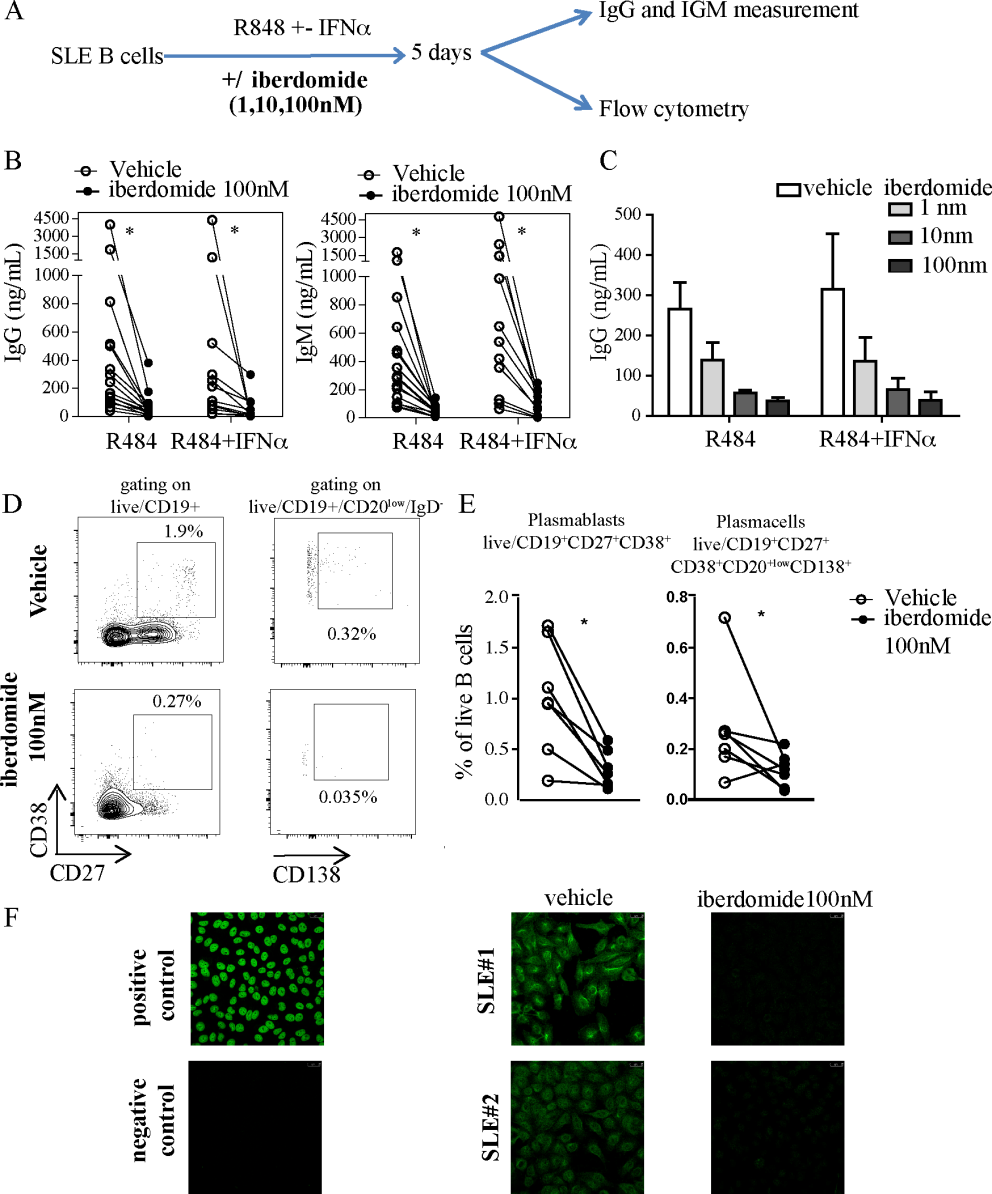
TLR7-induced and IFN-induced activation of SLE B cells. (A) Overview of experimental setup: B cells from SLE patients were triggered with TLR7 ligand R848 (Resiquimod) with iberdomide (1–10–100 nM) or vehicle as shown. After 5 days, cells were harvested and underwent flow-cytometry, while supernatants were used to measure ELISA. (B, C). IgG and IgM measured in the supernatants of cells stimulated as above with iberdomide at 100 nM (B) or 1–10–100 nM (C). (D) Representative dot-plots of flow cytometry after gating as shown and (E) cumulative results. (F) ANA measurement by IF in the supernatants of cells treated as in (A). n=16 patients with SLE in (B), 6 in (C), 7 in (E), with representative results in (D) and (F), p<0.05 Mann-Whitney in (B) and (E). ANA, antinuclear antibodies; SLE, systemic lupus erythematosus.

To explore the effects of therapeutic targeting of Ikaros and Aiolos on B cell activation in the context of SLE, we isolated B cells from blood samples from patients with SLE and stimulated them in vitro with TLR7 agonist resiquimod in combination with IFNα for 5 days, without or with the cereblon modulator iberdomide (figure 1A). TLR7 and IFNα stimulation of B cells promotes IgM^+^ response and expansion of autoreactive B cells^35^ and overactivity of both the TLR7 and IFNα system are established features of SLE.^36–38^ In our experiments, iberdomide significantly inhibited the production of IgG and IgM from B cells induced by resiquimod alone or resiquimod +IFNα (figure 1B), an effect that was shown to be dose-dependent, with both 10 and 100 nM inducing a significant reduction of Ig production compared with vehicle (figure 1C). Additionally, iberdomide induced a significant reduction in the percentage of CD19^+^CD27^+^CD38^+^ plasmablasts (iberdomide 0.3±0.18 vs vehicle 1.01±0.56, p=0.011) and CD20^low^CD27^+^CD38^+^CD138^+^ plasma cells (iberdomide 0.12±0.06 vs vehicle 0.28±0.02, p=0.03) (figure 1D, E). Iberdomide was able to abrogate the production of NA) induced by resiquimod +IFNα, as measured by HEp2 immunofluorescence (figure 1F). Overall, these data show that iberdomide inhibits TLR7-mediated activation and differentiation of SLE B cells and abrogates the production of ANA induced in autoreactive B cells by TLR7 and IFNα triggering.

### Iberdomide inhibits TLR7-mediated differentiation of plasmablasts from SLE B cells

**Figure 2:**
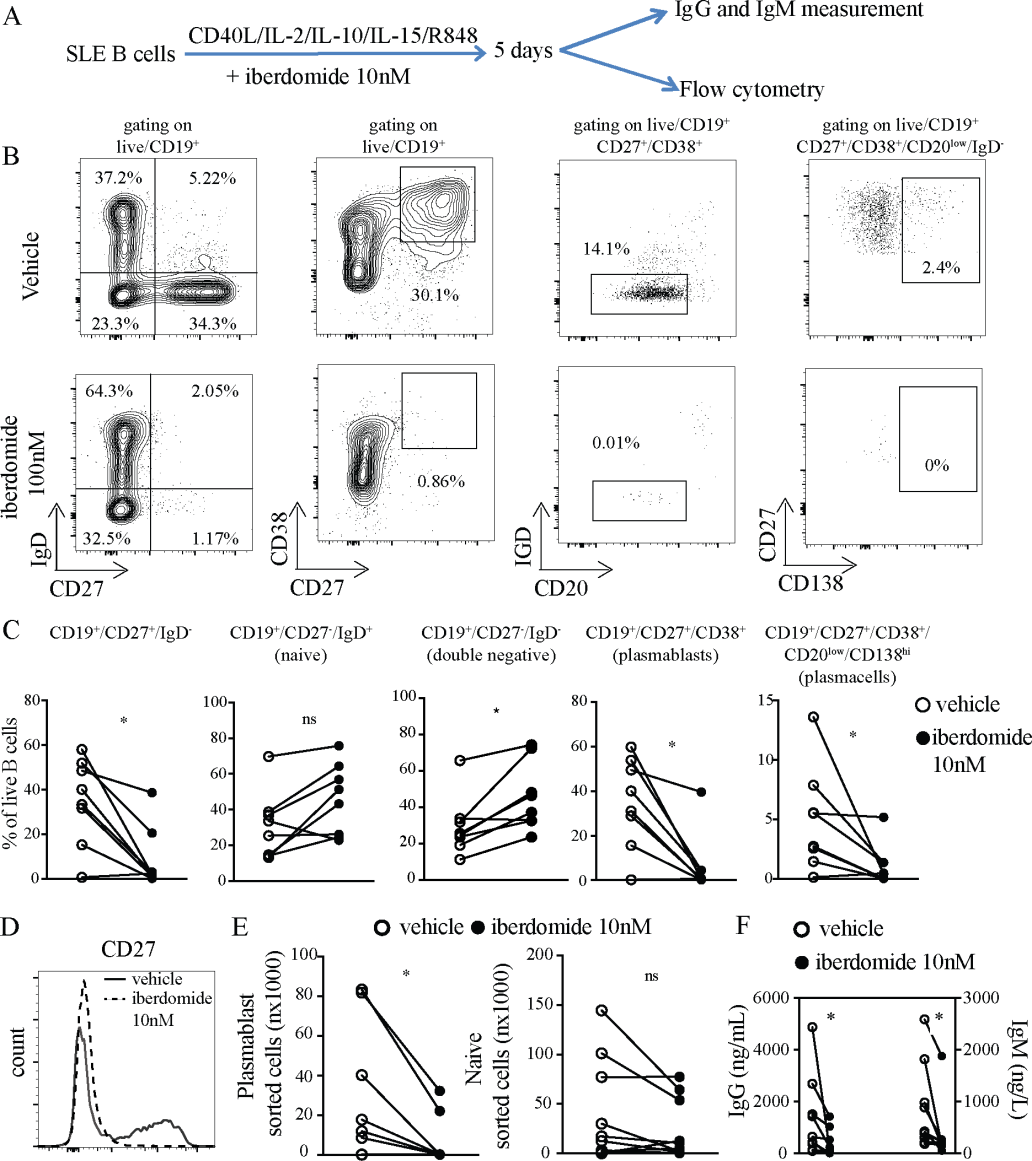
Differentiation of SLE B cells into plasmablasts. (A) B cells from patients with SLE were triggered with HA-sCD40L (50 ng/mL) cross-linked with anti-HA IgG (1 µg/mL), IL-2 (20 U/mL), IL-10 (50 ng/mL), IL-15 (10 ng/mL) and TLR7 ligand R848 (3 uM), plus Iberdomide (10 nM) or control vehicle from day 0. (B) Representative dot-plots of flow cytometry of cells harvested after 5 days of culture. Gating on live CD19+. (C) Cumulative results of flow cytometry (D) representative histogram of CD27 expression by B cells treated with iberdomide or vehicle. (E) Absolute numbers of plasmablasts (CD27+CD38+) and naïve B cells sorted after 5 days of culture as above. (F) IgG and IgM measured in the supernatants of cells treated as above. n=8 patients with SLE in (C), (E) and (F), with representative results in (B) and (D). P<0.05 Mann-Whitney in C-E-F. SLE, systemic lupus erythematosus.

In order to explore the effects of iberdomide on the differentiation of B cells in SLE, we adapted a previously described method for differentiating B cells into plasma cells, which includes stimulation with IL2, IL10, IL15 and CD40L, substituting TLR7 agonist Resiquimod instead of TLR9 agonist CpG. Iberdomide at 10 nM or vehicle were added as indicated (the experimental setup is summarised in figure 2A). The dose of 10 nM was chosen since the dose-response experiments presented in figure 1C showed a significant functional effect at this dose, which is closer to pharmacologically efficacious levels in vivo. Treatment of B cells with iberdomide resulted in a significant reduction in the percentage of CD27^+^IgD^-^ B cells (iberdomide 8.6±13.9 vs vehicle 34.9±19.3, p=0.028), without reducing the percentage of IgD^+^CD20^low^ naïve B cells—if anything, treatment with iberdomide showed a trend towards an increase in the percentage of naïve B cells (iberdomide 45.6±19.9 vs vehicle 30.7±18.9, p=0.160) and led to a significant increase in double negative (DN) CD27^-^IgD^-^ B cells considered part of the class-switched memory compartment (iberdomide 45.89±16.7 vs vehicle 29.4±16.2, p=0.015) (figure 2B, C). Additionally, iberdomide reduced the percentage of CD27^+^CD38^+^ plasmablasts (iberdomide 6.4±13.5 vs vehicle 34.9±20.1, p=0.013) and CD20^low^CD27^+^CD38^+^CD138^+^ plasma cells (iberdomide 1.0±1.7 vs vehicle 4.0±4.3, p=0.010) (figure 2B, C). Accordingly, we observed profound suppression of CD27 expression, as shown in the representative dot-plots in figure 2B and the histogram in figure 2D. When cells underwent FACS, the absolute numbers of sorted plasmablasts was significantly lower in iberdomide-treated samples (iberdomide 6,085±12 215 vs vehicle 27,123±33 888, p=0.014), while the number of sorted naïve B cells was numerically lower, but not statistically significantly different (iberdomide 23 647±31 910 vs vehicle 44 849±50 678, p=0.09) (figure 2E). Furthermore, supernatants collected from B cells cultured in the presence of iberdomide had significantly lower levels of secreted IgG (iberdomide 272±327 vs 1357±1565, p=0.02) and IgM (iberdomide 204±72 vs vehicle 891±811, p=0.001) compared with vehicle (figure 2F).

These results show that treatment with iberdomide significantly inhibits TLR7 mediated differentiation of SLE B cells into plasmablasts and plasma cells in vitro. As a consequence, antibody production is significantly reduced. Importantly, there were no significant differences in the percentage and absolute numbers of naïve B cells, suggesting a lack of impact on the overall survival of B cells, but rather a specific inhibition of their differentiation into plasmablasts.

### Effects of Iberdomide on differentiated plasmablasts

**Figure 3:**
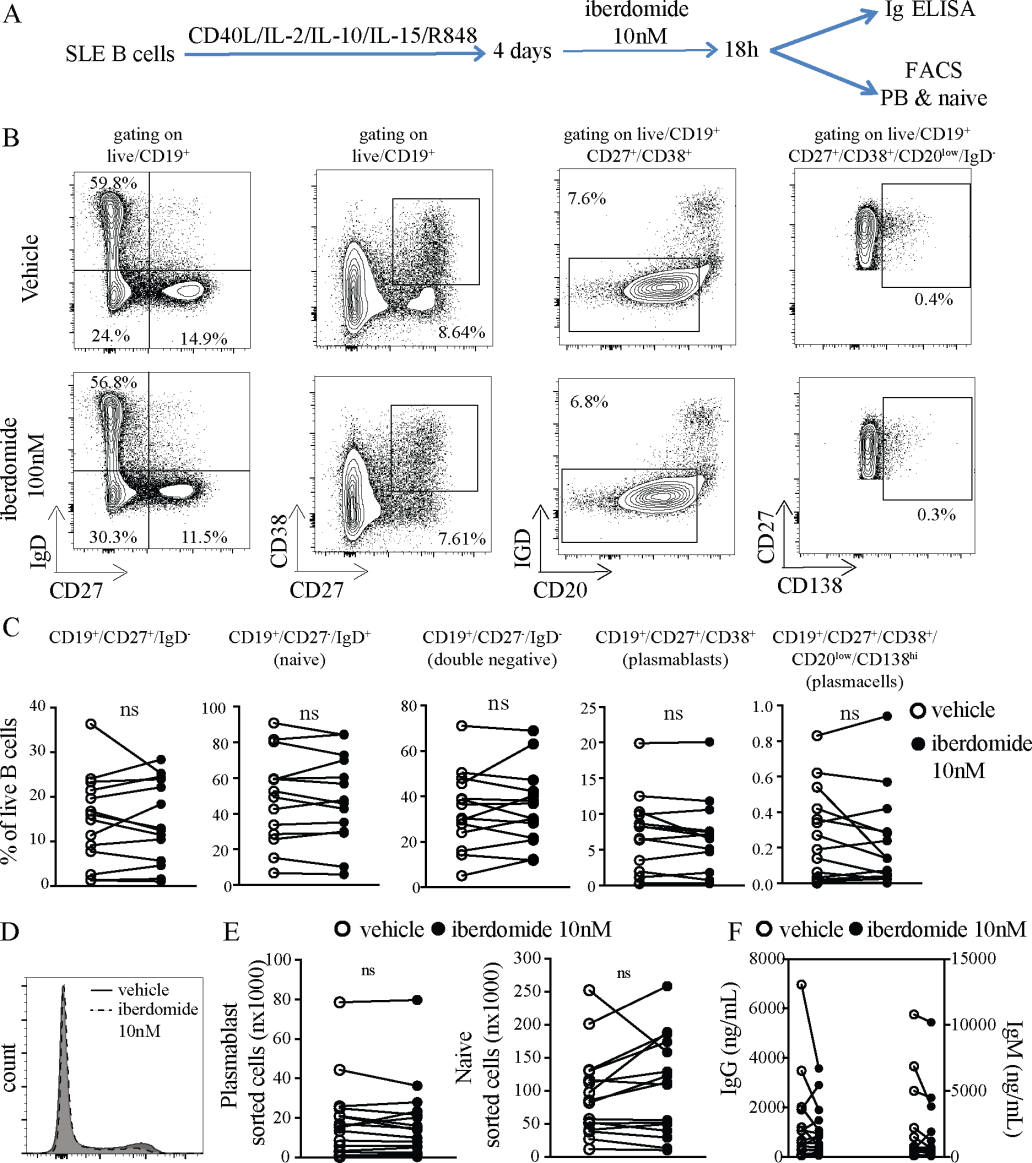
Modulation of SLE B cell differentiation. (A) B cells from patients with SLE were triggered with HA-sCD40L (50 ng/mL) cross-linked with anti-HA IgG (1 µg/mL), IL-2 (20 U/mL), IL-10 (50 ng/mL), IL-15 (10 ng/mL) and TLR7 ligand R848 (3 uM) for 4 days, then Iberdomide (10 nM) or control vehicle was added at day 4 for 18 hours. (B) Representative dot-plots of flow cytometry experiments with cells harvested after 5 days of culture. Gating on live CD19+. (C) Cumulative results of flow cytometry (D) representative histogram of CD27 expression by B cells treated with iberdomide or vehicle. (E) Absolute numbers of plasmablasts (CD27+CD38+) and naïve B cells (IgD+CD20 low) sorted after 5 days of culture as above. (F) IgG and IgM measured in the supernatants of cells treated as above. n=16 patients with SLE in C-F-G, with representative results in (B) and (D). P<0.05 Mann-Whitney test in C-E-F. SLE, systemic lupus erythematosus.

To investigate the effects of iberdomide-mediated IKZF1 and IKZF3 inhibition on the transcriptional profiles of SLE plasmablasts, SLE B cells were differentiated into plasmablasts using TLR7, IL2, IL10, IL15 and CD40L costimulation as before. However, since addition of iberdomide throughout the culture completely blocked B cell differentiation into plasmablasts, in order to dissect its effects on transcriptional programmes separate from apoptosis programmes, in the next set of experiments iberdomide was only added 18 hours prior to cell sorting (at day 4) as shown in figure 3A. Iberdomide added to SLE B cells on day 4 did not impact the percentage of CD27^+^IgD^-^ B cells, CD27^+^CD38^+^ plasmablasts, CD20^low^CD27^+^CD38^+^CD138^+^ plasma cells and naïve B cells (figure 3B, C) nor the expression of CD27 on B cells (figure 3D). Accordingly, the absolute numbers of sorted plasmablasts and naïve B cells were unaffected (figure 3E). Iberdomide given briefly on day 4 did not alter production of IgG and IgM (figure 3F). Overall, this shows that treatment of in vitro differentiated plasmablasts with iberdomide for a shorter time did not affect the percentage and absolute numbers of B cell subsets, thus enabling transcriptomic analysis of iberdomide-treated plasmablasts to assess which genes are immediately impacted downstream of co-inhibition of IKZF1 and IKZF3.

### Impact of Iberdomide on gene expression in naïve B cells and plasmablasts

**Figure 4:**
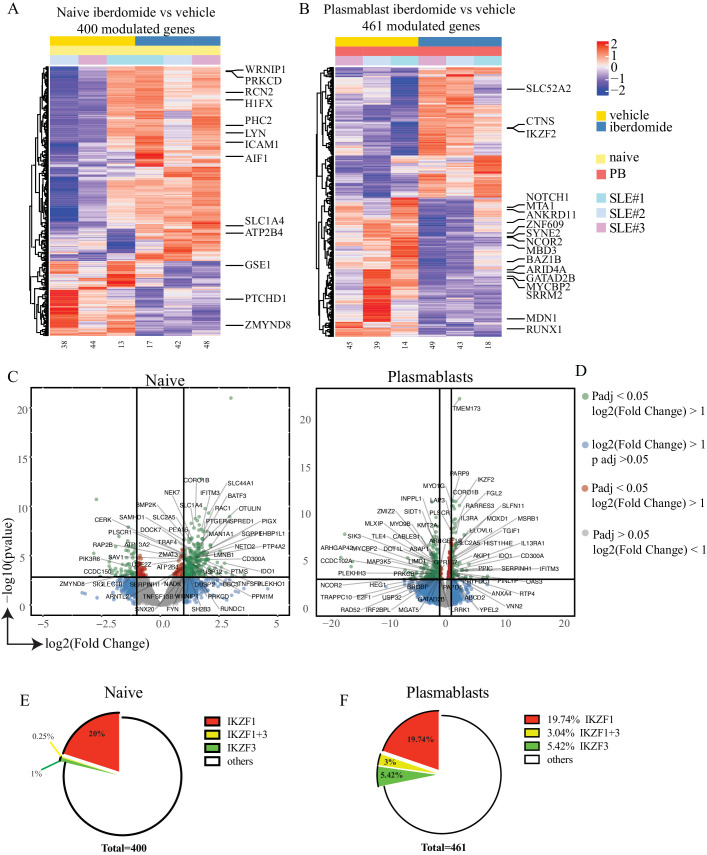
Modulation of gene expression by iberdomide. (A, B) Hierarchical clustering of differentially modulated genes from RNA sequencing of sorted naïve B cells (A) and plasmablasts (B) after treatment as shown in figure 3(A). (C, D) Volcano plots showing genes that are significantly modulated by iberdomide in naïve B cells (C) and plasmablasts (D). Labelling is showing genes known to be modulated by Ikaros and Aiolos. (E, F) percentage of the total genes modulated in naïve B cells (E) and plasmablasts (F) that are known to be targets of Ikaros and Aiolos or both. n=3 patients with SLE from three independent experiments. SLE, systemic lupus erythematosus.

Sorted naïve B cells and plasmablasts treated with iberdomide were subjected to RNA-sequencing to determine the direct influence of iberdomide on gene expression. Initial comparison of differentiated plasmablasts and naïve B cells showed an upregulation of *XBP1, IRF4, PRDM1* and the downregulation of *BACH2, CIITA, PAX5* and *BCL6,* consistently with previously reported results in plasma cell differentiation,^30^ thus indicating that the modified differentiation protocol effectively activated plasmablast transcriptional programming (online supplemental figure 2). Then, we assessed cells treated with iberdomide, and we found a number of genes significantly modulated by iberdomide both in naïve B cells (400 genes) and plasmablasts (461 genes). Figure 4A, B show the results of unsupervised hierarchical clustering of the differentially expressed genes, with labelling for genes known to be regulated by Ikaros and Aiolos (online supplemental table 1), according to published transcription factor binding site profiling studies^33^ and literature on Ikaros and Aiolos.^5 14 34^ Genes which were significantly upregulated or downregulated by iberdomide in naïve B cells and plasmablasts are shown in the volcano plot in figure 4C, D, again identifying multiple genes which are known targets of Ikaros and Aiolos. Geneset enrichment analysis using the Enrichr R package showed that the differentially genes were significantly associated with CHIP-Seq datasets for *IKZF1* (p=6.82×10^-9^) and *IKZF3* (p=0.0007) target genes. Overall, more than 20% of the differentially modulated genes were identified as known targets of Ikaros and Aiolos (figure 4E, F). Many of these genes are also known to be involved in the pathogenesis of SLE (eg, *NOTCH1, ZNF609, NCOR2, MYCBP2, SRRM2, MDN1, RUNX1, IGKV2D-10; IGLV3-17; IGHV1-45, CASP1, FGL2*). Additional gene set enrichment analysis using EnrichR^39^ was performed in order to identify pathways modulated by iberdomide using the Reactome pathway database. Analysis of the most significant pathways downregulated by iberdomide in plasmablasts (online supplemental figure 1) identified pathways involved in chromatin modification and organisation as well as cell signalling (Rho GTPase, G-alpha), apoptosis (NRAGE signals death through c-Jun N-terminal kinase (JNK)), cytokine signalling (IL-2, IL-3) and cell cycle (BMAL1:CLOCK, p75 NTR, beta-catenin complex, VEGFR mediated cell proliferation). Overall, these data suggest that treatment of SLE B cells with iberdomide significantly affects gene expression downstream of Ikaros and Aiolos, thus modulating a number of critical cellular survival pathways in differentiating B cells.

## Discussion

In this study, we investigated the effects of pharmacologically targeting the transcription factors Ikaros and Aiolos using the cereblon modulator iberdomide on B cells isolated from patients with SLE. First, when B cells were triggered with the TLR7 ligand Resiquimod and IFNα, iberdomide could inhibit their activation and antibody production, including the production of autoantibodies (ANA). Additionally, iberdomide abrogated the in vitro differentiation of B cells into plasmablasts induced by IL-2/IL-10/IL-15/CD40L/Resiquimod, a protocol for the differentiation of plasmablasts that we adapted to include triggering with TLR7 agonist instead.^30^ Finally, when added to already differentiated plasmablasts, iberdomide induced a significant modulation of gene expression both in naive B cells and plasmablasts. Overall, these data support targeting of Ikaros and Aiolos by iberdomide as a therapeutic strategy in SLE, because of its ability to inhibit B cell activation and differentiation.

This is in line with previously published data, showing that (i) iberdomide treatment in vitro could reduce Ikaros and Aiolos expression in SLE B cells and inhibit the differentiation of B cells into plasmablasts induced by IL-2/IL-21/BAFF^27^ and (ii) the administration of iberdomide to healthy volunteers could reduce Ikaros and Aiolos levels in immune cells and inhibit anti-dsDNA and antiphospholipid antibody production from SLE mononuclear cells.^28^ Similar results have been shown for another thalidomide analogue, lenalidomide, which inhibited the generation of preplasmablasts and early plasma cells, moderately affected plasmablast production and inhibited long-lived plasma cell generation, but did not impair their long-term survival once generated.^22^

In our experiments, we further explored the effects of iberdomide on B cell activation by triggering B cells with the TLR7 ligand Resiquimod. TLR7 is highly relevant for the pathogenesis of SLE because of its ability to recognise RNA, released as a consequence of defective clearance of apoptotic debris, which represents a key pathogenetic mechanism in SLE^40^ and other autoimmune diseases.^41^ Overactivity of the TLR7 and IFNα system are well-established features of SLE.^36–38^ Additionally, while different triggers, including also CD40L/IL21, are able to support B cell survival and proliferation in culture, only TLR7 and IFNα have been shown to promote IgM^+^ response and expansion of autoreactive B cells and production of ANA in otherwise non-autoreactive healthy B cells, as TLR7 and IFNα stimulation of B cells.^31^ Except for a few genetic studies linking polymorphisms of *IKZF1* and *IKZF3* to SLE,^42^ the exact function of Ikaros family of transcription factors in the development of autoimmunity remains to be clarified. Only one recent study has investigated the association of Ikaros family of transcription factors with TLR signalling.^15^ The authors demonstrated hyperactive TLR signalling accompanied by the inability to induce B cell receptor anergy in Ikaros-deficient mice, thus leading to a predisposition to autoimmunity. Interestingly, animals with germinal centre B cell-specific Ikaros deletion developed neither autoimmunity nor germinal centre B cells, indicating that the loss of Ikaros stringently blocked germinal centre B cell development. This points towards a double-faceted function of Ikaros in the development of autoimmunity: on one hand, Ikaros can suppress B cell auto-reactivity, thus ‘protecting’ against autoimmunity; on the other hand, it is also essential for the activation and differentiation of B cells, as confirmed by the observation that Ikaros-deficient mice lack circulating plasma cells. Interestingly, Ikaros-deficient mice had signs of systemic autoimmunity but did not develop autoimmune disease, suggesting that additional factors are needed for the development of full-blown autoimmunity. Similarly, coding mutations of *IKZF1* in humans mainly present with hypogammaglobulinaemia, while only a subset of patients develop autoimmunity.^43–46^ Overall, this suggests that Ikaros acts as a complex modulator of B cell activation and therefore represents a valuable target in autoimmune diseases. Accordingly, our experiments indicate that the addition of iberdomide throughout the whole duration of B cell culture was able to abrogate differentiation of B cells into plasmablasts and plasma cells, comparable to observations in Ikaros-deficient animals, which lack circulating plasma cells.

Importantly, iberdomide did not affect the overall survival of B cells, as the absolute number of naïve B cells and the percentage of naïve B cells did not change significantly and, if anything, higher levels of naïve B cells were found in iberdomide-treated patients, potentially a consequence of blockade of their differentiation. We observed a significant increase of the IgD-CD27- (DN) population on treatment with iberdomide. DN B cells,^47^ which are considered part of the memory compartment since they are class-switched, are expanded in SLE patients with high disease activity.^48^ Recent studies have shown that subclasses of DN B cells (DN2), which are TLR7 hyper-responsive, may promote SLE pathogenesis via their propensity to differentiate into plasmablasts following TLR7 activation.^49^ Overall, the relative increase in naïve B cells and DN B cells indicates that iberdomide is able to inhibit the TLR7 driven differentiation of B cells into plasmablasts.

These results consolidate previous data on B cells activated with CD40L and BAFF,^27^ providing further insights into the relevance of targeting Ikaros and Aiolos in patients with SLE because of the ability to inhibit TLR7 mediated B cell differentiation. The TLR7-driven differentiation protocol used in our study led to the expansion of both DN cells and plasmablasts in the absence of iberdomide. However, administration of iberdomide caused a drop in plasmablast number without affecting significantly naïve and DN B cells that were relatively increased. This observation supports the notion that iberdomide is not merely an anti-B cell compound, but through Ikaros and Aiolos downregulation, targets specifically plasmablast differentiation programming from naïve and DN cells, thus apparently halting the differentiation at that level. However, further studies are required to show whether iberdomide can block DN memory B cells expanded in SLE from differentiating into plasmablasts following TLR7 ligation. Although the lack of differences in the number of sorted naïve (IgD +CD27-) B cells clearly suggests that iberdomide does not impact B cell survival, a limitation of our study is that due to sample size and limited cell numbers we were not able to perform formal proliferation assays or use counting beads for additional accuracy.

Addition of iberdomide at a later stage to already differentiated plasma cells (18 hours before sorting), induced a modulation of gene expression both in naïve B cell and plasmablasts. This specific time-point was chosen based on previous time-course experiments, showing that treatment of B cells with iberdomide at 10 nM induced an inhibition of Ikaros and Aiolos lasting up to 24 hours.^28^ By performing RNA-sequencing of sorted naïve B cells and plasmablasts treated with iberdomide or control vehicle, we demonstrated that postactivation of the plasmablast transcriptional programme, iberdomide modulates a number of genes known to be regulated by Ikaros and Aiolos. Importantly, over 20% of genes that were modulated by iberdomide are known to be direct targets of Ikaros and Aiolos, in line with the known ability of iberdomide to induce the degradation of Ikaros and Aiolos.^26^ Although this is an exploratory analysis on a small number of patients, unsupervised hierarchical clustering identified a number of differentially expressed genes, many of which are known to be regulated by Ikaros/Aiolos, but also involved in the pathogenesis of SLE. For example, we identified: *NOTCH1*, whose blockade ameliorates SLE in animal models;^50^ *ZNF609*, a circular RNA implicated in lupus nephritis;^51^ *NCOR2*, relevant for the regulation of interferon production;^52^ *MYCBP2*, a newly described E3 ubiquitin-protein ligase;^53^ *SRRM2*, a splicing factor gene, with polymorphisms linked to SLE;^54^ *MDN1*, a human chaperone recently identified as a relevant factor in autoimmunity;^55^ *RUNX1*, a transcription factor associated with a number of autoimmune conditions, including rheumatoid arthritis, psoriasis and SLE.^56^

Similarly, analyses of differentially regulated genes in plasmablasts treated with iberdomide identified a number of genes known to be involved in the pathogenesis of SLE, such as immunoglobulin related genes (eg, *IGKV2D-10*; *IGLV3-17*; *IGHV1-45*); *CASP1*, encoding caspase-1, shown to be essential for the development of lupus in animal models;^57^ *FGL2*, fibrinogen-like 2, for which deletion leads to autoimmune glomerulonephritis.^58^

Additionally, pathway analysis identified a number of key cellular pathways that were downregulated by iberdomide in plasmablasts and may be important for plasmablast survival (inhibition of cell death signalling via JNK) during B cell differentiation and maturation and plasma cell survival in the bone marrow niche (beta catenin signalling). These data are consistent with previous known effects of targeting Ikaros and Aiolos leading to increased IL-2 signals. RNA-Seq analysis suggests that the inhibition of plasmablast/plasma cell differentiation and/or cell death of differentiating plasmablasts observed in our earlier experiments may be due to increased apoptosis in developing plasmablasts through increased death signals via JNK and alteration of cell cycle. Beta catenin is important for plasma cell survival via communication with bone marrow niche through syndecan-1 (also known as the plasma cell marker CD138),^59^ and so disruption of the beta catenin Wnt system may also lead to reduced plasma cell survival. Overall, these data indicate that iberdomide mediated degradation of Ikaros and Aiolos regulates key genes and pathways relevant for aberrant plasmablast differentiation during SLE pathogenesis.

These in vitro findings are also consistent with the recently reported clinical results for iberdomide in a SLE phase IIb study. Iberdomide showed significant efficacy in the treatment of active SLE, with enhanced effects observed in two biomarker-defined populations (Aiolos-High and Type 1 IFN-High), providing a rationale for this novel mechanism in SLE.^60^ In this trial, a significant decrease in anti-dsDNA antibodies was observed only after 24 weeks of iberdomide treatment.^61^ If there were a direct effect on plasmablast survival and immunoglobulin production, a more immediate decrease in anti-dsDNA antibodies would have been observed in this clinical trial. These clinical observations match the pattern of expression of cereblon, the molecular target of iberdomide, as it was found to be highly expressed in naïve B cells but downregulated once the differentiation in plasmablast is achieved.

In conclusion, we show that the cereblon modulator iberdomide inhibits the activation and differentiation of B cells and modulates the gene expression in differentiating plasmablasts from SLE patients, thus supporting the therapeutic targeting of Ikaros family transcription factors in SLE and potentially other autoimmune conditions.

## References

[1] Yoshida T, Georgopoulos K. Ikaros fingers on lymphocyte differentiation. Int J Hematol 2014;100:220–9. 10.1007/s12185-014-1644-5 25085254 PMC4492456

[2] Georgopoulos K, Bigby M, Wang JH, et al. The Ikaros gene is required for the development of all lymphoid lineages. Cell 1994;79:143–56. 10.1016/0092-8674(94)90407-3 7923373

[3] Georgopoulos K, Winandy S, Avitahl N. The role of the Ikaros gene in lymphocyte development and homeostasis. Annu Rev Immunol 1997;15:155–76. 10.1146/annurev.immunol.15.1.155 9143685

[4] Kirstetter P, Thomas M, Dierich A, et al. Ikaros is critical for B cell differentiation and function. Eur J Immunol 2002;32:720. 10.1002/1521-4141(200203)32:3&lt;720::AID-IMMU720&gt;3.0.CO;2-P 11870616

[5] Schwickert TA, Tagoh H, Gültekin S, et al. Stage-specific control of early B cell development by the transcription factor Ikaros. Nat Immunol 2014;15:283–93. 10.1038/ni.2828 24509509 PMC5790181

[6] Morgan B, Sun L, Avitahl N, et al. Aiolos, a lymphoid restricted transcription factor that interacts with Ikaros to regulate lymphocyte differentiation. Embo J 1997;16:2004–13. 10.1093/emboj/16.8.2004 9155026 PMC1169803

[7] Wang JH, Avitahl N, Cariappa A, et al. Aiolos regulates B cell activation and maturation to effector state. Immunity 1998;9:543–53. 10.1016/S1074-7613(00)80637-8 9806640

[8] Han J-W, Zheng H-F, Cui Y, et al. Genome-wide association study in a Chinese Han population identifies nine new susceptibility loci for systemic lupus erythematosus. Nat Genet 2009;41:1234–7. 10.1038/ng.472 19838193

[9] Cunninghame Graham DS, Morris DL, Bhangale TR, et al. Association of NCF2, IKZF1, IRF8, IFIH1, and Tyk2 with systemic lupus erythematosus. PLoS Genet 2011;7:e1002341. 10.1371/journal.pgen.1002341 22046141 PMC3203198

[10] Gateva V, Sandling JK, Hom G, et al. A large-scale replication study identifies TNIP1, PRDM1, Jazf1, UHRF1BP1 and IL10 as risk loci for systemic lupus erythematosus. Nat Genet 2009;41:1228–33. 10.1038/ng.468 19838195 PMC2925843

[11] Lessard CJ, Adrianto I, Ice JA, a IJ, et al. Identification of IRF8, TMEM39A, and IKZF3-ZPBP2 as susceptibility loci for systemic lupus erythematosus in a large-scale multiracial replication study. Am J Hum Genet 2012;90:648–60. 10.1016/j.ajhg.2012.02.023 22464253 PMC3322228

[12] Cai X, Qiao Y, Diao C, et al. Association between polymorphisms of the IKZF3 gene and systemic lupus erythematosus in a Chinese Han population. PLoS One 2014;9:e108661–11. 10.1371/journal.pone.0108661 25271777 PMC4182708

[13] Zhang Y-M, Zhou X-J, Cheng F-J, et al. Association of the IKZF1 5' UTR variant rs1456896 with lupus nephritis in a northern Han Chinese population. Scand J Rheumatol 2017;46:210–4. 10.1080/03009742.2016.1194458 27684961

[14] Westra H-J, Peters MJ, Esko T, et al. Systematic identification of trans eQTLs as putative drivers of known disease associations. Nat Genet 2013;45:1238–43. 10.1038/ng.2756 24013639 PMC3991562

[15] Schwickert TA, Tagoh H, Schindler K, et al. Ikaros prevents autoimmunity by controlling anergy and Toll-like receptor signaling in B cells. Nat Immunol 2019;20:1517–29. 10.1038/s41590-019-0490-2 31591571 PMC7115902

[16] Celhar T, Magalhães R, Fairhurst A-M. Tlr7 and TLR9 in SLE: when sensing self goes wrong. Immunol Res 2012;53:58–77. 10.1007/s12026-012-8270-1 22434514

[17] Chamberlain PP, Lopez-Girona A, Miller K, et al. Structure of the human Cereblon-DDB1-lenalidomide complex reveals basis for responsiveness to thalidomide analogs. Nat Struct Mol Biol 2014;21:803–9. 10.1038/nsmb.2874 25108355

[18] Gandhi AK, Kang J, Havens CG, et al. Immunomodulatory agents lenalidomide and pomalidomide co-stimulate T cells by inducing degradation of T cell repressors Ikaros and Aiolos via modulation of the E3 ubiquitin ligase complex CRL4(CRBN.). Br J Haematol 2014;164:811–21. 10.1111/bjh.12708 24328678 PMC4232904

[19] Krönke J, Udeshi ND, Narla A, et al. Lenalidomide causes selective degradation of IKZF1 and IKZF3 in multiple myeloma cells. Science 2014;343:301–5. 10.1126/science.1244851 24292625 PMC4077049

[20] Lu G, Middleton RE, Sun H, et al. The myeloma drug lenalidomide promotes the cereblon-dependent destruction of Ikaros proteins. Science 2014;343:305–9. 10.1126/science.1244917 24292623 PMC4070318

[21] Attal M, Lauwers-Cances V, Marit G, et al. Lenalidomide maintenance after stem-cell transplantation for multiple myeloma. N Engl J Med 2012;366:1782–91. 10.1056/NEJMoa1114138 22571202

[22] Jourdan M, Cren M, Schafer P, et al. Differential effects of lenalidomide during plasma cell differentiation. Oncotarget 2016;7:28096–111. 10.18632/oncotarget.8581 27057635 PMC5053712

[23] Okon L, Rosenbach M, Krathen M, et al. Lenalidomide in treatment-refractory cutaneous lupus erythematosus: efficacy and safety in a 52-week trial. J Am Acad Dermatol 2014;70:583–4. 10.1016/j.jaad.2013.11.007 24528907 PMC4333148

[24] Braunstein I, Goodman NG, Rosenbach M, et al. Lenalidomide therapy in treatment-refractory cutaneous lupus erythematosus: histologic and circulating leukocyte profile and potential risk of a systemic lupus flare. J Am Acad Dermatol 2012;66:571–82. 10.1016/j.jaad.2011.01.015 21821308 PMC3306524

[25] Cortés-Hernández J, Ávila G, Vilardell-Tarrés M, et al. Efficacy and safety of lenalidomide for refractory cutaneous lupus erythematosus. Arthritis Res Ther 2012;14:R265. 10.1186/ar4111 23217273 PMC3674591

[26] Matyskiela ME, Zhang W, Man H-W, et al. A cereblon modulator (CC-220) with improved degradation of Ikaros and Aiolos. J Med Chem 2018;61:535–42. 10.1021/acs.jmedchem.6b01921 28425720

[27] Nakayama Y, Kosek J, Capone L, et al. Aiolos overexpression in systemic lupus erythematosus B cell subtypes and BAFF-Induced memory B cell differentiation are reduced by CC-220 modulation of cereblon activity. J Immunol 2017;199:2388–407. 10.4049/jimmunol.1601725 28848067 PMC5602157

[28] Schafer PH, Ye Y, Wu L, et al. Cereblon modulator iberdomide induces degradation of the transcription factors Ikaros and Aiolos: immunomodulation in healthy volunteers and relevance to systemic lupus erythematosus. Ann Rheum Dis 2018;77:1516–23. 10.1136/annrheumdis-2017-212916 29945920 PMC6161670

[29] Petri M, Orbai A-M, Alarcón GS, et al. Derivation and validation of the systemic lupus international collaborating clinics classification criteria for systemic lupus erythematosus. Arthritis Rheum 2012;64:2677–86. 10.1002/art.34473 22553077 PMC3409311

[30] Jourdan M, Caraux A, De Vos J, et al. An in vitro model of differentiation of memory B cells into plasmablasts and plasma cells including detailed phenotypic and molecular characterization. Blood 2009;114:5173–81. 10.1182/blood-2009-07-235960 19846886 PMC2834398

[31] Rouillard AD, Gundersen GW, Fernandez NF, et al. The harmonizome: a collection of processed datasets gathered to serve and mine knowledge about genes and proteins. Database 2016;2016. 10.1093/database/baw100. [Epub ahead of print: 03 Jul 2016]. PMC4930834 27374120

[32] ENCODE Project Consortium. The encode (encyclopedia of DNA elements) project. Science 2004;306:636–40. 10.1126/science.1105136 15499007

[33] Lachmann A, Xu H, Krishnan J, et al. Chea: transcription factor regulation inferred from integrating genome-wide ChIP-X experiments. Bioinformatics 2010;26:2438–44. 10.1093/bioinformatics/btq466 20709693 PMC2944209

[34] Ferreirós-Vidal I, Carroll T, Taylor B, et al. Genome-wide identification of Ikaros targets elucidates its contribution to mouse B-cell lineage specification and pre-B-cell differentiation. Blood 2013;121:1769–82. 10.1182/blood-2012-08-450114 23303821

[35] Simchoni N, Cunningham-Rundles C. TLR7- and TLR9-responsive human B cells share phenotypic and genetic characteristics. J Immunol 2015;194:3035–44. 10.4049/jimmunol.1402690 25740945 PMC4369401

[36] Marshak-Rothstein A, Rifkin IR. Immunologically active autoantigens: the role of Toll-like receptors in the development of chronic inflammatory disease. Annu Rev Immunol 2007;25:419–41. 10.1146/annurev.immunol.22.012703.104514 17378763

[37] Bennett L, Palucka AK, Arce E, et al. Interferon and granulopoiesis signatures in systemic lupus erythematosus blood. J Exp Med 2003;197:711–23. 10.1084/jem.20021553 12642603 PMC2193846

[38] Garcia-Romo GS, Caielli S, Vega B, et al. Netting neutrophils are major inducers of type I IFN production in pediatric systemic lupus erythematosus. Sci Transl Med 2011;3:73ra20–73. 10.1126/scitranslmed.3001201 PMC3143837 21389264

[39] Chen EY, Tan CM, Kou Y, et al. Enrichr: interactive and collaborative HTML5 gene list enrichment analysis tool. BMC Bioinformatics 2013;14:128. 10.1186/1471-2105-14-128 23586463 PMC3637064

[40] Tsokos GC, Lo MS, Costa Reis P, et al. New insights into the immunopathogenesis of systemic lupus erythematosus. Nat Rev Rheumatol 2016;12:716–30. 10.1038/nrrheum.2016.186 27872476

[41] Saferding V, Blüml S. Innate immunity as the trigger of systemic autoimmune diseases. J Autoimmun 2020;110:102382. 10.1016/j.jaut.2019.102382 31883831

[42] Teruel M, Alarcón-Riquelme ME. The genetic basis of systemic lupus erythematosus: what are the risk factors and what have we learned. J Autoimmun 2016;74:161–75. 10.1016/j.jaut.2016.08.001 27522116

[43] Kuehn HS, Boisson B, Cunningham-Rundles C, et al. Loss of B cells in patients with heterozygous mutations in Ikaros. N Engl J Med 2016;374:1032–43. 10.1056/NEJMoa1512234 26981933 PMC4836293

[44] Hoshino A, Okada S, Yoshida K, et al. Abnormal hematopoiesis and autoimmunity in human subjects with germline IKZF1 mutations. J Allergy Clin Immunol 2017;140:223–31. 10.1016/j.jaci.2016.09.029 27939403

[45] Bogaert DJ, Kuehn HS, Bonroy C, et al. A novel Ikaros haploinsufficiency kindred with unexpectedly late and variable B-cell maturation defects. J Allergy Clin Immunol 2018;141:432–5. 10.1016/j.jaci.2017.08.019 28927821 PMC6588539

[46] Van Nieuwenhove E, Garcia-Perez JE, Helsen C, et al. A kindred with mutant Ikaros and autoimmunity. J Allergy Clin Immunol 2018;142:699–702. 10.1016/j.jaci.2018.04.008 29705243 PMC6541477

[47] Sanz I, Wei C, Jenks SA, et al. Challenges and opportunities for consistent classification of human B cell and plasma cell populations. Front Immunol 2019;10. 10.3389/fimmu.2019.02458 PMC6813733 31681331

[48] Wei C, Anolik J, Cappione A, et al. A new population of cells lacking expression of CD27 represents a notable component of the B cell memory compartment in systemic lupus erythematosus. J Immunol 2007;178:6624–33. 10.4049/jimmunol.178.10.6624 17475894

[49] Jenks SA, Cashman KS, Zumaquero E, et al. Distinct effector B cells induced by unregulated Toll-like receptor 7 contribute to pathogenic responses in systemic lupus erythematosus. Immunity 2018;49:725–39. 10.1016/j.immuni.2018.08.015 30314758 PMC6217820

[50] Zhang W, Xu W, Xiong S. Blockade of Notch1 signaling alleviates murine lupus via blunting macrophage activation and M2b polarization. J Immunol 2010;184:6465–78. 10.4049/jimmunol.0904016 20427764

[51] Luan J, Jiao C, Kong W, et al. circHLA-C plays an important role in lupus nephritis by sponging miR-150. Mol Ther Nucleic Acids 2018;10:245–53. 10.1016/j.omtn.2017.12.006 29499937 PMC5768151

[52] Kim TW, Hong S, Lin Y, et al. Transcriptional repression of IFN regulatory factor 7 by Myc is critical for type I IFN production in human plasmacytoid dendritic cells. J Immunol 2016;197:3348–59. 10.4049/jimmunol.1502385 27630164 PMC5101139

[53] Pao K-C, Wood NT, Knebel A, et al. Activity-based E3 ligase profiling uncovers an E3 ligase with esterification activity. Nature 2018;556:381–5. 10.1038/s41586-018-0026-1 29643511

[54] Pullabhatla V, Roberts AL, Lewis MJ, et al. De novo mutations implicate novel genes in systemic lupus erythematosus. Hum Mol Genet 2018;27:421–9. 10.1093/hmg/ddx407 29177435 PMC5886157

[55] Shooshtari P, Huang H, Cotsapas C. Integrative genetic and epigenetic analysis uncovers regulatory mechanisms of autoimmune disease. Am J Hum Genet 2017;101:75–86. 10.1016/j.ajhg.2017.06.001 28686857 PMC5501874

[56] Alarcón-Riquelme ME. Role of Runx in autoimmune diseases linking rheumatoid arthritis, psoriasis and lupus. Arthritis Res Ther 2004;6:169–73. 10.1186/ar1203 15225361 PMC464920

[57] Kahlenberg JM, Yalavarthi S, Zhao W, et al. An essential role of caspase 1 in the induction of murine lupus and its associated vascular damage. Arthritis Rheumatol 2014;66:152–62. 10.1002/art.38225 24449582 PMC4135431

[58] Shalev I, Liu H, Koscik C, et al. Targeted deletion of fgl2 leads to impaired regulatory T cell activity and development of autoimmune glomerulonephritis. J Immunol 2008;180:249–60. 10.4049/jimmunol.180.1.249 18097026

[59] Ren Z, van Andel H, de Lau W, et al. Syndecan-1 promotes Wnt/β-catenin signaling in multiple myeloma by presenting Wnts and R-spondins. Blood 2018;131:982–94. 10.1182/blood-2017-07-797050 29212806

[60] Merrill J, Werth V, Furie R. Efficacy and Safety of Iberdomide in Patients with Active Systemic Lupus Erythematosus: 24-Week Results of a Phase 2, Randomized, Placebo-Controlled Study [abstract]. Arthritis Rheumatol 2020;72. [Epub ahead of print: March 11, 2021] https://acrabstracts.org/abstract/efficacy-and-safety-of-iberdomide-in-patients-with-active-systemic-lupus-erythematosus-24-week-results-of-a-phase-2-randomized-placebo-controlled-study/

[61] Lipsky P, Vollenhoven Rvan, Dörner T, et al. Iberdomide decreases B cells and plasmacytoid dendritic cells, increases regulatory T cells and IL-2, and has enhanced clinical efficacy in active systemic lupus erythematosus patients with high Aiolos or the IFN gene expression signature. arthritis Rheumatol, 2020. Available: https://acrabstracts.org/abstract/iberdomide-decreases-b-cells-and-plasmacytoid-dendritic-cells-increases-regulatory-t-cells-and-il-2-and-has-enhanced-clinical-efficacy-in-active-systemic-lupus-erythematosus-patients-with-high-aiolo/

