# Supplementary material for: Effects of targeting the transcription factors Ikaros and Aiolos on B cell activation and differentiation in systemic lupus erythematosus
Source: Lupus Sci Med. 2021 Mar 16;8(1):e000445. doi: 10.1136/lupus-2020-000445 (PMC7970264; doi:10.1136/lupus-2020-000445)
Supplement: Supplementary data [file lupus-2020-000445supp001.pdf]

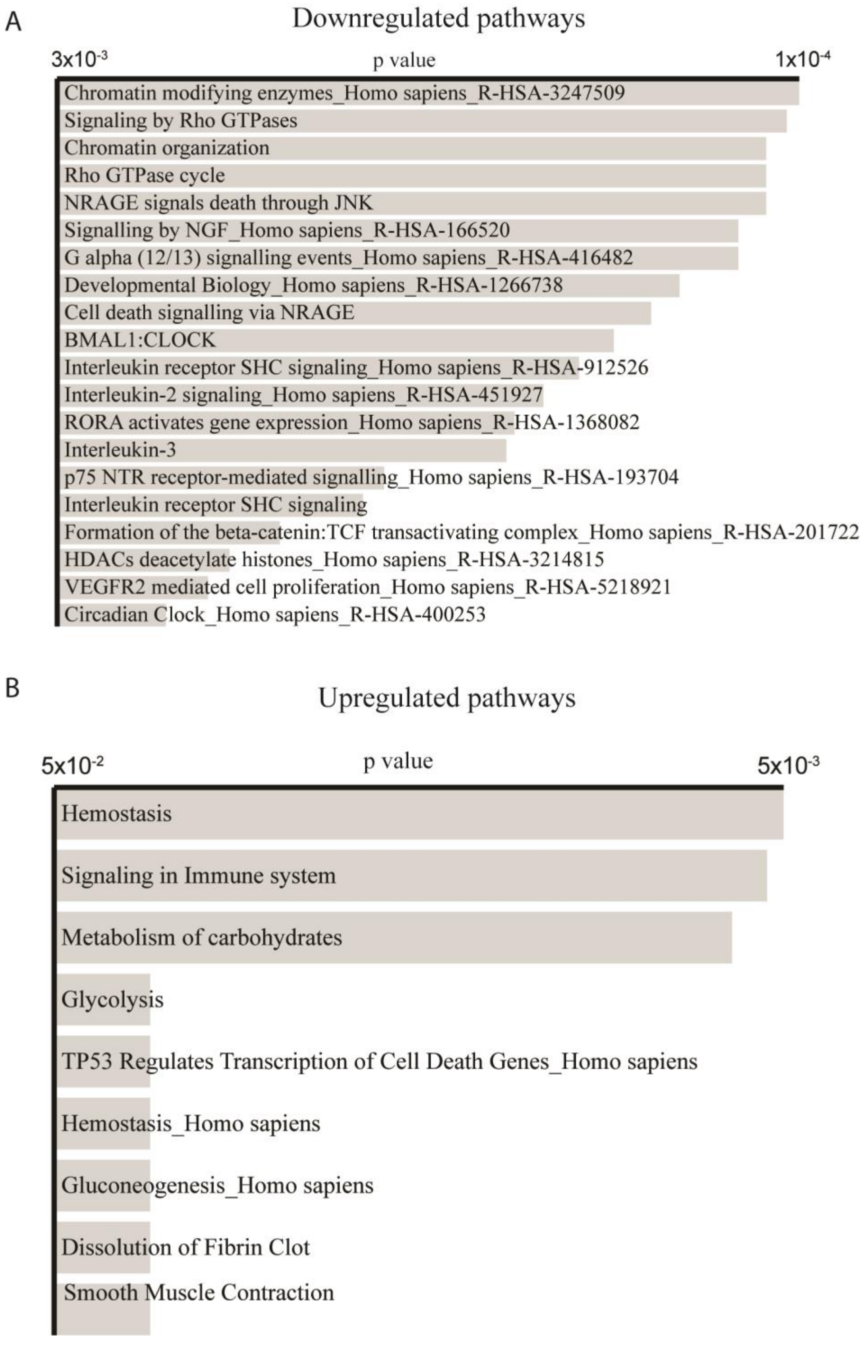

Supplementary figure 1. Pathways modulated in plasmablasts by iberdomide

A-B Pathway analysis by EnrichR, showing the most significant modulated pathways down-regulated (A) and up-regulated (B) in plasmablasts, ordered according to adjusted p values

Supplementary table 1: FACS Antibodies

Target	Clone	Catalogue no.	Company	Fluorophore	Dilution	Laser channel
CD20	2H7	45020942	eBioscience	PerCP-Cy5.5	1:80	B 695/40
CD19	HIB19	302242	Biolegend	BV510	1:80	V 520/10
CD38	HIT 2	303528	Biolegend	BV711	1:40	V 710/50
CD138	MI15	356516	Biolegend	BV421	1:40	V 450/50
CD27	O323	17027942	eBioscience	APC	1:80	R 670/14
IgD	IA6-2	348204	Biolegend	PE	1:40	Y/G 582/15
CD3	HIT3A	300306	Biolegend	FITC	1:300	B 530/30
CD14	M5E2	301804	Biolegend	FITC	1:80	B 530/30
CD56	HCD56	318304	Biolegend	FITC	1:80	B 530/30
Zombie	NA	423106	Biolegend	NIR	1:170	R780/60

Rivellese F, *et al. Lupus Sci Med* 2021; 8:e000445. doi: 10.1136/lupus-2020-000445

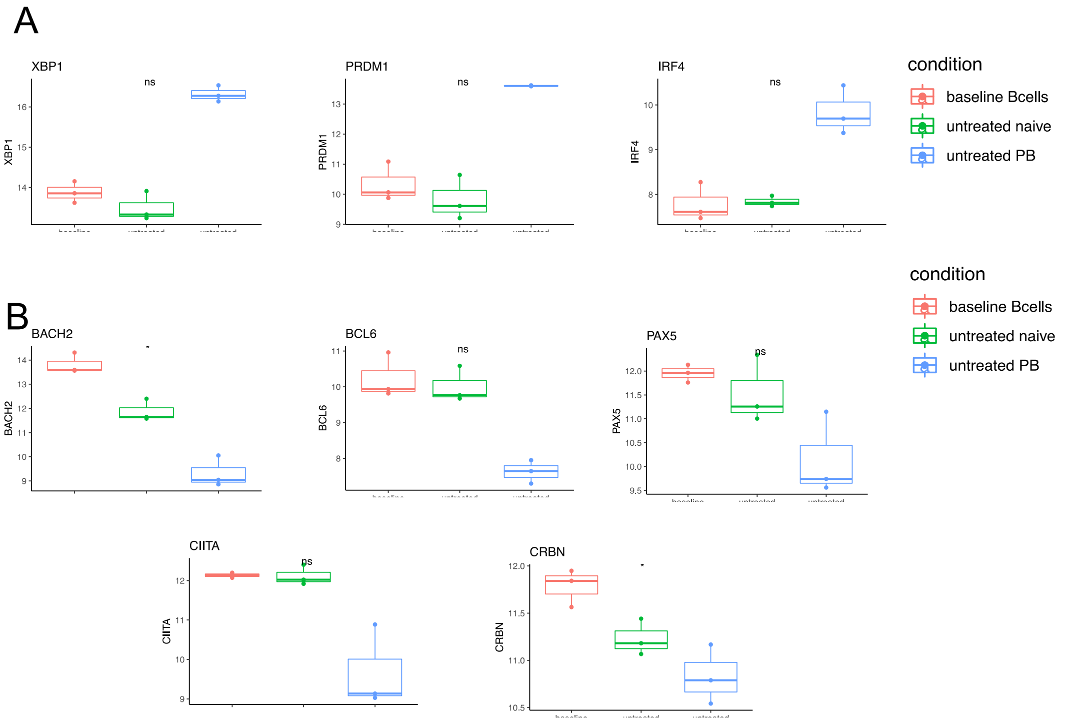

Supplementary figure 2. Plasma cell genes in B cells after isolation (baseline) and -plasmablasts (CD27+CD38+) and naïve B cells (IgD+CD20low) sorted after 5 days of culture with HA-sCD40L (50 ng/mL) cross-linked with anti-HA IgG (1 µg/mL), IL-2 (20 U/mL), IL-10 (50 ng/mL), IL-15 (10 ng/mL) and TLR7 ligand R848 (3 uM). Genes upregulated in plasmacells are shown in A and downregulated in B. n=3 SLE patients. *p<0.05 by Kruskal-Wallis.

Rivellese F, *et al. Lupus Sci Med* 2021; 8:e000445. doi: 10.1136/lupus-2020-000445
